# G × EBLUP: A novel method for exploring genotype by environment interactions and genomic prediction

**DOI:** 10.3389/fgene.2022.972557

**Published:** 2022-09-12

**Authors:** Hailiang Song, Xue Wang, Yi Guo, Xiangdong Ding

**Affiliations:** ^1^ Beijing Key Laboratory of Fisheries Biotechnology, Fisheries Science Institute, Beijing Academy of Agriculture and Forestry Sciences, Beijing, China; ^2^ Key Laboratory of Animal Genetics and Breeding of the Ministry of Agriculture and Rural Affairs, National Engineering Laboratory for Animal Breeding, College of Animal Science and Technology, China Agricultural University, Beijing, China

**Keywords:** G × E interaction, snps, bayes factors, traits, genomic prediction

## Abstract

Genotype by environment (G × E) interaction is fundamental in the biology of complex traits and diseases. However, most of the existing methods for genomic prediction tend to ignore G × E interaction (GEI). In this study, we proposed the genomic prediction method G × EBLUP by considering GEI. Meanwhile, G × EBLUP can also detect the genome-wide single nucleotide polymorphisms (SNPs) subject to GEI. Using comprehensive simulations and analysis of real data from pigs and maize, we showed that G × EBLUP achieved higher efficiency in mapping GEI SNPs and higher prediction accuracy than the existing methods, and its superiority was more obvious when the GEI variance was large. For pig and maize real data, compared with GBLUP, G × EBLUP showed improvement by 3% in the prediction accuracy for backfat thickness, while our findings indicated that the trait of days to 100 kg of pig was not affected by GEI and G × EBLUP did not improve the accuracy of genomic prediction for the trait. A significant advantage was observed for G × EBLUP in maize; the prediction accuracy was improved by ∼5.0 and 7.7% for grain weight and water content, respectively. Furthermore, G × EBLUP was not influenced by the number of environment levels. It could determine a favourable environment using SNP Bayes factors for each environment, implying that it is a robust and useful method for market-specific animal and plant breeding. We proposed G × EBLUP, a novel method for the estimation of genomic breeding value by considering GEI. This method identified the genome-wide SNPs that were susceptible to GEI and yielded higher genomic prediction accuracies and lower mean squared error compared with the GBLUP method.

## Introduction

Genomic selection (GS) (Meuwissen et al., 2001), which relies on linkage disequilibrium between single nucleotide polymorphisms (SNPs) and causative variants, has become a useful tool in animal (VanRaden et al., 2009) and plant (Zhong et al., 2009) breeding. However, GS analytical modelling usually assumes no G × E interaction (GEI) and opposes the true genetic architecture of complex traits. In fact, interaction is fundamental in biology, and there is growing interest in estimating breeding value by considering GEI and using genome-wide SNPs.

The current state-of-the-art methods for the estimation of genomic breeding value without considering GEI include GBLUP (VanRaden, 2008) and Bayes-Alphabet (e.g., Bayes A, Bayes B and Bayes C) (Habier et al., 2011). Multi-trait (Richard, 1996) and reaction norm models (Rebecka et al., 2002) are the two prevalent GEI-handling methods that are used for genomic evaluations. However, the multi-trait model could only capture GEI in a limited number of environments, and the computational demands of multi-trait models would increase rapidly with an increase in the number of environment levels (Song et al., 2020a). The reaction norm model captures only part of the GEI because it needs to accommodate a continuous range of environmental values and cannot select excellent individuals using the unique estimated breeding value in actual breeding (Jarquin et al., 2014; Song et al., 2020b).

To explore GEI, certain G × E interaction-affected methods have been proposed for detecting SNPs. Wang et al. (2019) proposed several methods (Bartlett, F-Killeen, L-mean and L-median) that can be used to infer GEI from variance quantitative trait locus (vQTL) analysis without requiring environmental factor measurements. Moore et al. (2019) proposed StructLMM, which is useful for studying interactions with hundreds of environment variables. Moreover, Kerin and Marchini. (2020) proposed LEMMA, which infers GEI using a Bayesian whole-genome regression model. However, in Wang’s method, GEI-affected SNP detection is possible because of selection, epistasis and phantom vQTL, instead of only GEI (Wang et al., 2019). StructLMM and LEMMA do not currently enable accounting for relatedness, and these methods cannot be efficiently applied to livestock and plant breeding due to the close genetic relationships that widely exists between individuals (Kerin et al., 2020; Moore et al., 2019). Therefore, it is essential to develop new methods for the estimation of genomic breeding value by considering GEI.

In this study, we proposed a novel approach for the estimation of genomic breeding value by considering GEI, which can handle environment variables in different dimensions. The basic principle of the new approach was to first detect the genome-wide markers affected by GEI (G × EWAS), in which a score-test statistics was implemented to identify the significant GEI-associated SNPs. Next, all markers were classified into SNPs with/without GEI to construct the genomic relationship matrices separately and predict the genomic breeding value using the mixed model (G × EBLUP). For its general application, the efficiency of the proposed method was evaluated through simulation study and real data from pigs and maize.

## Methods

### Ethics statement

The animal study was reviewed and approved by Animal handling and sample collection were conducted according to protocols approved by the Institutional Animal Care and Use Committee (IACUC) at China Agricultural University. All authors strictly complied with the Regulations on the Administration of Laboratory Animals (Order No. 2 of the State Science and Technology Commission of the People’s Republic of China, 1988). There was no use of human participants, data or tissues.

### G × EWAS

G × EWAS extends the conventional linear mixed model by including an additional per-individual effect term accounting for G × E, which can be represented as N × 1 vector, $\beta_{GxE}$ . The model was defined as follows:

$$y=Xb+x\beta_{G}+x⊙\beta_{GxE}+u+e$$ (1)

where **y** is the vector of observed phenotypic values; b is the vector of fixed effects; $\beta_{G}$ is the average effect of gene substitution of a particular SNP; and x is the vector of the genotype indicator variable of the variant coded as 0, 1 or 2. $x⊙\beta_{G\times E}=diag\left(x\right)\beta_{G\times E}$ , where ⊙ denotes the element-wise (Hadamard) product and diag(x) denotes the N×N diagonal matrix whose diagonal is x .The per-individual effect size vector $\beta_{G\times E}$ is defined as a random effect, following the multivariate normal distribution $\beta_{G\times E}∼N\left(0,\;\sigma_{G\times E}^{2}\sum \right)$ , where $\sigma_{G\times E}^{2}$ , is the variance and covariance matrix of the G × E effect, $\sum \in R^{N\times N}$ parameterises how per-individual effects covary across individuals and is calculated as a function of observed environment variables. $\sum \equiv \sum \left(E\right)={EE}^{\prime }$ , where E is the N×L matrix of L observed environments. The linear covariance function ( ${EE}^{\prime }$ ) was primarily used because of two appealing properties. First, as the number of samples typically exceeds the number of environments in larger populations (L << N), a low-rank linear covariance is noted, which enables parameter inference with a computational complexity that scales linearly with the increasing population size. Second, a linear covariance is directly interpretable as there is one-to-one correspondence between G × EWAS and linear regression using L covariates to account for GEI. Notably, for the special case of $\sigma_{G\times E}^{2}=0$ , the model G × EWAS reduces to a standard linear mixed model for genome-wide association study; thus, G × EWAS is a single-SNP regression model; u is the vector of random polygenic effects with a normal distribution $u∼N\left(0,\;G\sigma_{u}^{2}\right)$ , in which $\sigma_{u}^{2}$ is the polygenic variance and G is the genomic relationship matrix. It was constructed using the markers according to VanRaden (2008); X is the incidence matrix linking b to **y**; e is the vector of random errors with normal distribution of N (0, **I** $\sigma_{e}^{2}$ ), where $\sigma_{e}^{2}$ is the residual variance and I is the identity matrix. The analysis of G × EWAS was based only on the reference data to avoid the double counting of the SNP effect in genomic prediction.

For the parameter inference, we considered the marginalised form of the model in Eq. 1, which was obtained by integrating over the G × E effects $\beta_{G\times E}$ and the random effect component u **:**

$$y∼N\left(Xb+x\beta_{G},\;\sigma_{GxE}^{2}diag\left(x\right){EE}^{T}diag\left(x\right)+\sigma_{g}^{2}G+\sigma_{e}^{2}I\right)$$ (2)

Using the marginalised model in Eq. 2, a G × E interaction test corresponds to the alternative hypothesis $\sigma_{G\times E}^{2}>0$ . We defined an efficient score-based test that enabled the *p*-value calculation with a complexity that scaled linearly with the number of individuals, provided that there is low-rank environment covariance ∑ . The null model of the interaction test reduced to a standard linear mixed model with a low-rank covariance matrix for additive genetic effects, and the existing efficient inference strategies for the standard linear mixed model can be reused. The score-test statistics can be computed in an analogous manner according to the procedure described by Wu et al. (2011):

$$Q=\frac{1}{2}y^{T}{PK}_{1}Py=\frac{1}{2}y^{T}P\left(\text{diag}\left(x\right){EE}^{T}\text{diag}\left(x\right)\right)Py=\frac{1}{2}y^{T}P\left(diag\left(x\right)E\right){\left(diag\left(x\right)E\right)}^{\prime }Py=\frac{1}{2}{\left|\left|W^{T}Py\right|\right|}^{2}$$ (3)

Where

$$K_{1}=\text{diag}\left(x\right)\sum \text{diag}\left(x\right)$$

W=diag(x)E

$$P=H_{0}^{-1}-H_{0}^{-1}\left[X,x\right]{\left({\left[X,x\right]}^{T}H_{0}^{-1}\left[X,x\right]\right)}^{-1}{\left[X,x\right]}^{T}H_{0}^{-1}$$

The matrix $H_{0}$ denotes the total covariance matrix estimated under the null model $H_{0}$ = $\sigma_{g}^{2}G$ **+** $\sigma_{e}^{2}I$ **Q** follows a mixture of $\chi^{2}$ distributions (Wu et al., 2011; Lippert et al., 2014): $Q∼\sum_{k}a_{k}\chi_{1}^{2}$ , where the vector of the coefficients $a={\left[a_{k}\right]}_{k}$ can be computed as the eigenvalues of $P^{\frac{T}{2}}K_{1}P^{\frac{1}{2}}$ . According to the procedure in SKAT (Wu et al., 2011), as the distribution of the score-test statistics was a mixture of $\chi^{2}$ , the *p*-values were computed using the Davies method (Davies, 1980). Alternatively, the Liu method (Huan et al., 2008) was employed when the Davies method failed to converge.

The evidences for individual environment variables or environment sets for driving the observed G × E effects can be assessed by comparing the model log marginal likelihoods between models with and without including these environments. The Bayes factors (BF) obtained from such comparisons is directly calibrated as the parameter number fitted using maximum likelihood and is independent of the environment variable numbers.

Given a variant and set of L environment L = ( $e_{1},e_{2},e_{3},\ldots ,e_{L}$ ),

$${(\mathrm{Log}\left(BF\right)=LML\left(L\right)-LML(L}_{i})$$ (4)

where LML(L) and LML( $L_{i}$ ) represent the marginal log-likelihood of the model described in Eq. 2, either considering the full or reduced environment sets to define the G × E environment covariance, respectively. log(BF) < 0 indicates the lack of contribution of the environmental impact on G × E interaction, whereas log(BF) > 3 indicates strong G × E environment interaction (Kass and Raftery., 1995).

### G × EBLUP

The G × EBLUP model includes additive genetic and GEI effects. The model is as follows:

$$y=Xb+{Zu}_{G\times E}+Zu+e$$ (5)

where y **,** X **,** b and e denote the same parameters as in the G × EWAS model, $u_{G\times E}$ is the vector of genomic values captured by genetic markers associated with GEI, following a normal distribution of $N(0,G_{G\times E}\sigma_{G\times E}^{2}$ ); u is the vector of genomic values captured by the remaining genetic marker sets (SNPs that are not significantly associated with GEI), following a normal distribution $N(0,\;G_{u}\sigma_{u}^{2}$ ) and Z is an incidence matrix that links $u_{G\times E}$ and u to y . Matrices $G_{G\times E}$ and $G_{u}$ were constructed similarly as G ; the former was constructed using only the genetic marker set defined by GEI, as described below, and the latter was constructed using the remaining markers. $\sigma_{G\times E}^{2}$ and $\sigma_{u}^{2}$ are the variance components explained by the variants with and without GEI, respectively. When an SNP was significant the GEI with phenotypes based on the prespecified significance cutoff level (E01-E05), showing the SNP was considered to impact the GEI.

### Data simulation

So far, only few genomic data simulating softwares considering GEI have been available. In this study, we proposed a reaction norm model accounting for heterogeneous residual variances to simulate phenotypic and environmental values.

$$y=\alpha_{0}+\alpha_{1}*c+e_{0}+e_{1}*c$$

where y is the vector of phenotypic value, c is the vector of environmental value; $\alpha_{0}$ and $\alpha_{1}$ are the random additive genetic effects for the intercept and slope, respectively; and $e_{0}$ and $e_{1}$ are the random residual effects for the intercept and slope, respectively.

The environmental value c is further divided into two components:

c=β+ϵ

where β is the vector of the random genetic effect and ε is the vector of the random residual effect.

We assumed that $\alpha_{0}$ , β and $\alpha_{1}$ are affected by all QTLs simultaneously, and these three effects of each QTL are drawn from a multivariate normal distribution with the vector of means 0 and the variance–covariance structure $\left[\begin{array}{ccc} \sigma_{\alpha_{0}}^{2} & \sigma_{\alpha_{0}\beta } & \sigma_{\alpha_{0}\alpha_{1}}\\ \sigma_{\alpha_{0}\beta } & \sigma_{\beta }^{2} & \sigma_{\beta \alpha_{1}}\\ \sigma_{\alpha_{0}\alpha_{1}} & \sigma_{\beta \alpha_{1}} & \sigma_{\alpha_{1}}^{2} \end{array}\right]$ . The genetic variance of each QTL is computed using 2 $p_{i}\left(1-p_{i}\right)m_{i}$ , where $p_{i}$ is the frequency of one allele of *i*th QTL, $m_{i}$ is the effect of the *i*th QTL for $\alpha_{0}$ , β or $\alpha_{1}$ . Then, the substitution effects are rescaled to ensure the total variances $\sigma_{\alpha_{0}}^{2}$ , $\sigma_{\beta }^{2}$ and $\sigma_{\alpha_{1}}^{2}$ for $\alpha_{0}$ , β and $\alpha_{1}$ , respectively. The $\sigma_{\alpha_{0}\beta }$ , $\sigma_{\alpha_{0}\alpha_{1}}$ and $\sigma_{\beta \alpha_{1}}$ are re-calculated using the scaled substitution effects of QTL. The $e_{0}$ , $e_{1}$ and ε values of each individual are sampled from a multivariate normal distribution with the vector of means 0 and the variance–covariance structure $\left[\begin{array}{ccc} \sigma_{e_{0}}^{2} & \sigma_{e_{0}e_{1}} & \sigma_{e_{0}\varepsilon }\\ \sigma_{e_{0}e_{1}} & \sigma_{e_{1}}^{2} & \sigma_{e_{1}\varepsilon }\\ \sigma_{e_{0}\varepsilon } & \sigma_{e_{1}\varepsilon } & \sigma_{\varepsilon }^{2} \end{array}\right]$ .

For the G × E interaction simulations, the parameter $\sigma_{\alpha_{1}}^{2}$ was set to control the extent of the G × E interaction, whereas other parameters ( $\sigma_{\alpha_{0}}^{2}$ , $\sigma_{\beta }^{2}$ , $\sigma_{\alpha_{0}\beta }$ , $\sigma_{\alpha_{0}\alpha_{1}}$ , $\sigma_{e_{0}}^{2}$ , $\sigma_{\beta \alpha_{1}}$ , $\sigma_{e_{1}}^{2}$ , $\sigma_{\epsilon }^{2}$ , $\sigma_{e_{0}e_{1}}$ , $\sigma_{e_{0}\epsilon }$ and $\sigma_{e_{1}\epsilon }$ ) were fixed. The pseudo true breeding values (TBVs) of an individual for $\alpha_{0}$ , β and $\alpha_{1}$ are its QTL effects multiplied by genotypes, followed by the scaling of the means of the pseudo TBVs to 0. Finally, the environmental valuec of each individual is obtained by adding the cumulative effect across all QTLs for β with the residual ϵ , followed by the generation of the phenotype y of each individual through the model $y=\alpha_{0}+\alpha_{1}*c+e_{0}$ , without accounting for heterogeneous residual variance. For the simulated data, c was used as the environment variable **E**, as described in formula (1). The real genotypes of 7,334 individuals determined using the Illumina BovineSNP50 BeadChip from the Chinese Holstein population were referred for phenotype and environment simulation, and 45,323 SNPs remained after imputation of missing genotypes and removal of SNPs with a minor allele frequency (MAF) of <0.01. Additional File 3 Supplementary Figure S1 presents the heat map of the genomic relationship matrix of 7,334 Chinese Holsteins. Three simulated datasets with GEI effect variances of 0.25, 1 and 2 were obtained, and the corresponding phenotypic variances were 2.25, 3 and 4, respectively. Additionally, when the GEI effect variance was 0.25, the datasets 2 and 3 covariate environments were simulated and compared. For each dataset, 306 SNPs that affected the trait of interest were simulated and referred to as simulated QTLs in this study. For each scenario, the simulation was repeated 20 times. We used the DMU software (Madsen et al., 2006) to estimate the variances of the additive effect, GEI effect and residual using the reaction norm model for each replicate. As shown in Additional File 2 Supplementary Table S1, these estimated values were close to the assigned values. Moreover, a dataset was randomly selected from the 20 repeated datasets, and 6 SNPs with GEI were randomly selected from this dataset, showing that the phenotypic variation was largely affected by GEI and that it was relatively small in the scenarios with no GEI (Additional File 3: Supplementary Figures S2–S6), thus implying that the simulation fitted well. All analyses with G × EBLUP and GBLUP models and simulation were conducted using in-house scripts written in Python3.8 by the first author.

### Real data

#### Pig data

Yorkshire pigs were sampled from a breeding company with five breeding farms distributed across China (Additional File 3: Supplementary Figure S7). Different farms displayed distinct climates, housing systems, nutritional regimes, disease pressures and stocking densities, potentially leading to GEI. Table 1 presents the phenotype data. We examined two growth traits ‘days to 100 kg (AGE)’ and ‘backfat thickness adjusted to 100 kg (BFT)’. Genotyping was performed using the PorcineSNP80 BeadChip (Illumina, CA, USA), which included 68,528 SNPs across the entire pig genome. A total of 1,778 animals born between 2011 and 2016 were genotyped (Table 1).

**TABLE 1:** Descriptive statistics for pig and maize population traits.

**Population**	**Trait** ^a^	**N-obs** ^b^	**Genotyped individuals**	**N-env** ^c^	**Mean**	**SD**	**Min**	**Max**
Pig	AGE (day)	28,827	1778	5	170.8	13.9	124.0	211.0
	BFT (mm)	28,827	1778	5	11.8	2.4	5.0	30.7
Maize	GW (kg)	2676	681	11	6.75	1.39	0.407	11.24
	WC (%)	2676	681	11	26.89	4.58	14.80	47.80

a AGE: days to 100 kg; BFT: backfat thickness adjusted to 100 kg; GW: grain weight; WC: water content.

b N-obs: number of observations.

c N-env: number of environments.

### Pig data

Maize is one of the most important crops worldwide. It provides food for humans and animals; it is a raw material for industrial processes and a model plant for understanding evolution, domestication and heterosis (Romay et al., 2013). Thus, maize data from 11 regions across China (Additional File 3: Supplementary Figure S7) were obtained to verify G × EBLUP, with the regions used as environment variables. Because of varying conditions of light, temperature, air, water and soil in different regions, under which GEI could show its effect, region effect was considered as an environmental covariate in the present study. A total of 681 maize lines were collected and each line had phenotype records of two traits, grain weight (GW) and water content (WC), in 1–8 environments. Overall, 2,676 observations were collected for the two traits. Table 1 lists the detailed information on GW and WC. Meanwhile, all lines were genotyped using the customised SNP panel of 61,224 markers across the maize genome.

For real pig and maize data, Beagle 4.1 (Browning and Browning, 2009) was used for the imputation of the missing SNP genotypes, and only loci on autosomes were used for further analysis. PLINK software (v1.90) (Chang et al., 2015) was implemented for quality control. We excluded SNPs with a MAF of <0.05, call rate of <0.90, or those severely deviating from the Hardy–Weinberg equilibrium (HWE) (*p* < 10^–7^). Similarly, we excluded the pig individuals or maize samples with a call rate of <0.90. Finally, 56,463 and 59,401 SNPs were present in the pig and maize data, respectively, and all genotyped pigs and maize were retained.

### Method application

#### Application to simulated data

Simulated data analysis was performed using G × EWAS proposed in this study to identify markers associated with GEI. We used Bonferroni correction at a significance level of 0.05 to identify significant SNPs. We implemented the four methods (L-median, L-mean, Bartlett and F-Killeen) proposed by Wang et al. (2019) in addition to StructLMM proposed by Moore et al. (2019) to identify SNPs affected by GEI. Based on the G × EWAS results, we performed genomic prediction on each simulated dataset using G × EBLUP. To investigate more SNPs with GEI, *p*-value gradients of 1-E01–1-E05 were chosen as threshold standards to select the SNPs associated with GEI. Five 10-fold cross-validation (10-CV) repetitions were used to assess the genomic prediction using G × EBLUP. In each cross-validation, the reference and validation populations comprised 6,601 and 733 individuals, respectively. The accuracy of genomic prediction was calculated as the Pearson’s correlation between original phenotypes Phe and the genomic estimated breeding values (GEBVs) of all validation individuals *r*(Phe, GEBV). Moreover, the mean squared error (MSE) of the prediction ability matrix was used to evaluate the performance of the models; MSE was computed as the average square of the difference between Phe and GEBV centred on zero. In each scenario, we performed the comparisons between G × EBLUP and GBLUP (VanRaden, 2008) at different GEI variances (0.25, 1 and 2) and different number of environment variables (1, 2 and 3).

#### Application to real data

Pig data were used to detect the SNPs affected by GEI. The herd-year-season effects, estimated using the conventional pedigree-based BLUP method, were used as environmental covariates, and the corrected phenotype were used as response variables. The calculations for the corrected phenotype value followed the method described by Song et al. (2017). Overall, 207 young and the remaining 1,571 individuals were considered as the validation and reference populations, respectively. For the maize data, an environment was randomly selected for each line to ensure that all lines could be used for analysis. Accordingly, we used 681 lines for each analysis and performed five replications of 5-fold cross-validation.

For the G × EBLUP method, the *p*-values for all markers were calculated using G × EWAS, and a threshold standard with *p*-value gradients of 1-E01–1-E05 was selected to screen the GEI-associated SNPs. The GBLUP and G × EBLUP methods were then used to estimate GEBVs. The genomic prediction accuracy on the real data was evaluated differently than that on the simulated data, using the correlation between GEBVs and the phenotypic values y (maize data) or corrected phenotypes y_c_ (pig data) in the validation population. MSE was computed as the average square of the difference between y or y_c_ and GEBVs centred on zero. The BF for each environment variable was calculated to obtain the sensitive environments of markers associated with GEI. For simulated data and real data, the improvement in prediction accuracy for G × EBLUP over GBLUP was calculated by subtracting the prediction accuracy obtained by GBLUP from the prediction accuracy obtained by G × EBLUP and then dividing by the prediction accuracy obtained by GBLUP.

## Results

### Genome-wide G × E association analysis

#### Simulated data

Table 2 indicates that G × EWAS performed better than the other methods. When the GEI variance was 0.25, G × EWAS detected 2,435 significant SNPs with Bonferroni correction (0.05/45,293). Of these SNPs, 43 overlapped with the 306 simulated GEI SNPs. Although a low number of SNPs (41) overlapped with the simulated GEI SNPs, StructLMM detected a high number of significant SNPs (2,472). Similarly, G × EWAS detected a higher number of significant SNPs than those detected using the Bartlett, F-Killeen, L-mean and L-median methods, which identified 507, 101, 224 and 188 significant SNPs, respectively. Further, 9, 6, 6 and 6 SNPs overlapped with the simulated GEI QTLs, respectively. A similar trend was also observed in the scenario where GEI variance increased to 1 or 2. Larger GEI effect variances led to the identification of a higher number of real QTLs with GEI (with the exception of the F-Killeen method), as shown in Table 2 and Additional File 3 Supplementary Figures S8–S10.

**TABLE 2:** Significant G × E interaction single nucleotide polymorphisms (SNPs) detected on simulated data using the proposed G × EWAS method and the five approaches, StructLMM, Bartlett, F-Killeen, L-mean and L-median under different variance of G × E interactions. The SNP numbers overlapping with simulated genotype–environment interaction quantitative trait locus (306) are in parentheses.

Variance of G × E interactions	0.25	1	2
G × EWAS	2435 (43)	5081 (64)	3981 (77)
StructLMM	2472 (41)	5092(62)	4084 (77)
Bartlett	507 (9)	3495 (33)	3976 (54)
F-killeen	101 (6)	144 (2)	109 (3)
L-mean	224 (6)	1037 (8)	606 (14)
L-median	188 (6)	808 (8)	439 (9)

#### Pig and maize data

**FIGURE 1:**
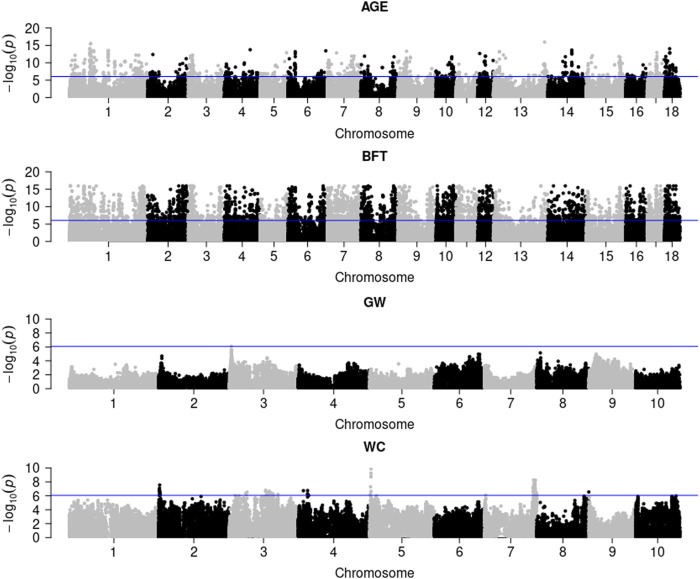
G × E marker genome-wide association analysis of pig and maize population traits. AGE: days to 100 kg; BFT: backfat thickness adjusted to 100 kg; GW: grain weight; WC: water content.

Figure 1 illustrates the genome-wide G × E marker mapping on pig and maize data. Figure 1 shows that a total of 1,164 and 5,448 significant SNPs were detected in pigs for AGE and BFT traits with Bonferroni correction (0.05/56,445), respectively. For maize data, only 1 and 84 genome-wide significant SNPs were detected for the two traits, GW and WC, respectively. Additionally, the genotypic values and SNP effect values of the top most significant SNPs for each trait at different environments in pig and maize populations indicated that BFT, GW and WC were affected by the environment; however, no GEI was detected on AGE (Additional File 3: Supplementary Figures S11, S12).

### Accuracy and mean squared error of genomic prediction

#### Simulated data

**FIGURE 2:**
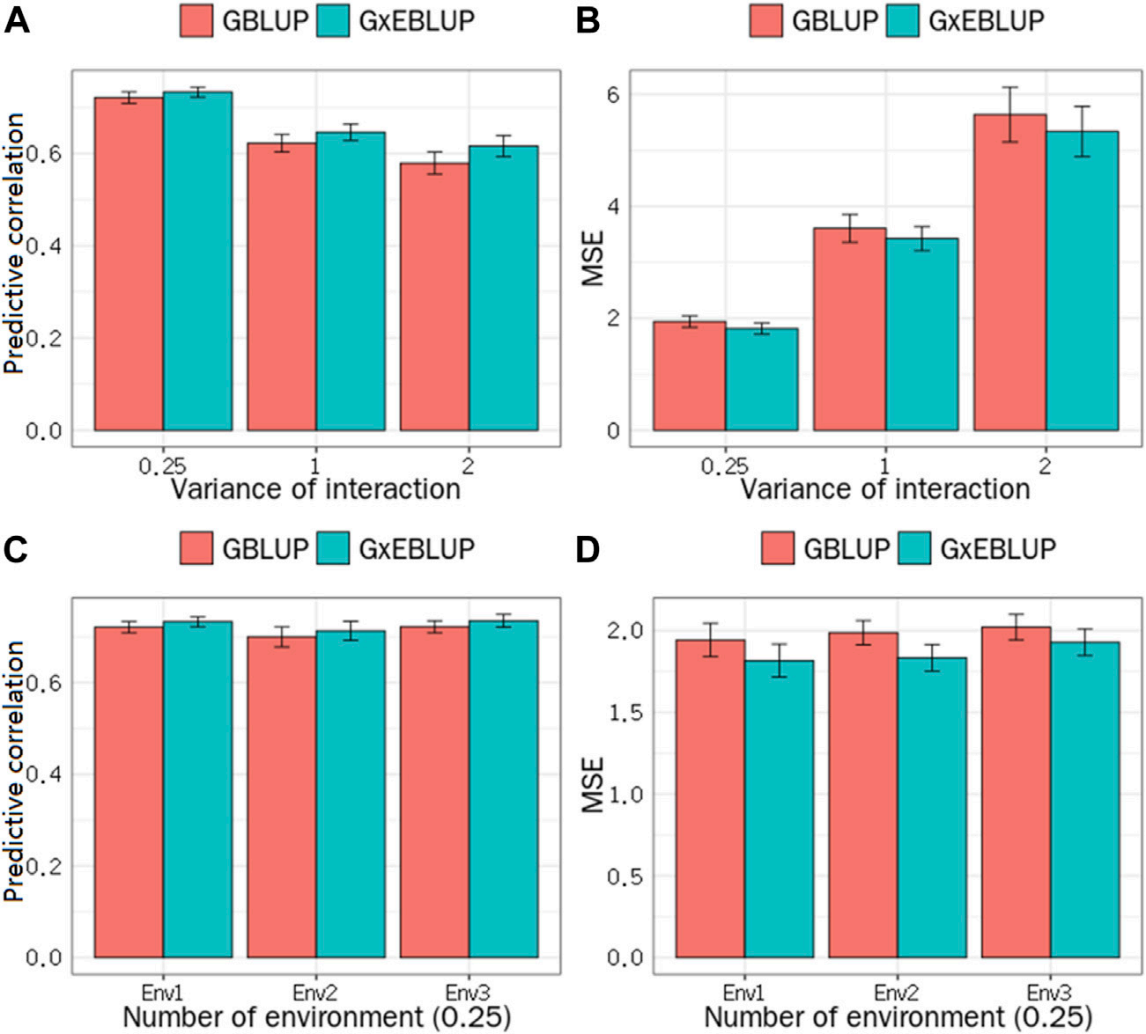
GBLUP and G × EBLUP method for the **(A)** accuracy and **(B)** mean squared error (MSE) of genomic prediction under different G × E interaction variances. Genomic prediction **(C)** accuracy and **(D)** MSE under different numbers of environment variables.

The significance level was set at the *p*-value gradient of 1-E01–1-E05 to determine SNPs associated with GEI. Table 3 presents the number of SNPs affected by GEI. These SNPs along with the corresponding remaining SNPs were used in G × EBLUP. All 45,323 qualified SNPs were used in GBLUP. The genomic prediction accuracy and MSE of the simulated data were determined from a randomly selected replicate. Table 3 shows that the G × EBLUP accuracy was different under different *p*-values. G × EBLUP showed the highest genomic prediction accuracy and the lowest MSE with a *p*-value of 1-E03. Further, the prediction accuracy of G × EBLUP improved by 1.7% compared with that of GBLUP. Figure 2 shows the averaged accuracies and MSE of genomic prediction obtained using G × EBLUP and GBLUP under different GEI variances. For G × EBLUP, the average prediction accuracy was calculated by selecting the highest prediction accuracy values under different *p*-values in each repetition. When the GEI variance was 0.25, the G × EBLUP yielded 1.7% higher prediction accuracies than GBLUP. Moreover, G × EBLUP yielded lower MSE than GBLUP, with average MSE values of 1.816 and 1.942, respectively. G × EBLUP performed significantly better than GBLUP when the GEI variance was increased to 1 and 2, yielding 3.9 and 6.4% higher prediction accuracies, respectively, and a lower MSE than GBLUP.

**TABLE 3:** Genomic prediction accuracies and mean squared error (MSE) for GBLUP and G × EBLUP method under different G × E interaction *p*-values (E01∼E05).

Data set	Trait	Content	GBLUP	P-value^a^				
				E01	E02	E03	E04	E05
Simulation^b^	One^c^	SNP number	45,323	23,517	14,210	8844	5543	2186
		Accuracy	0.737	0.735	0.739	0.749	0.738	0.712
		MSE	1.818	1.820	1.810	1.715	1.813	1.894
Pig	AGE	SNP number	56,445	27,762	14,117	7420	3964	2117
		Accuracy	0.225	0.223	0.226	0.226	0.226	0.224
		MSE	179.81	179.918	179.722	179.703	179.677	179.797
	BFT	SNP number	56,445	37,448	25,242	17,110	11,801	8098
		Accuracy	0.268	0.275	0.276	0.272	0.269	0.268
		MSE	2.707	2.693	2.69	2.699	2.704	2.706
Maize^b^	GW	SNP number	59,401	17,285	4279	834	421	143
		Accuracy	0.288	0.290	0.306	0.294	0.271	0.269
		MSE	46.323	46.317	46.174	46.178	46.223	46.347
	WC	SNP number	59,401	28,636	12,123	4168	2132	875
		Accuracy	0.295	0.301	0.315	0.318	0.293	0.273
		MSE	721.588	721.229	721.129	720.854	721.590	722.009

a Cut-off p-values for G × E interaction single nucleotide polymorphisms on G × E.

b One randomly selected replicate.

c The variance of G × E interactions was 0.25.

Figure 2 also shows how the number of the environment variables influences the genomic prediction. In the scenario with a GEI variance of 0.25, when the number of environment variables was 1, 2 and 3, no significant differences were noted in the prediction accuracy and MSE among the number of different environment variables for G × EBLUP. Additionally, in all scenarios, G × EBLUP yielded 1.8% higher accuracies and a 12.4% lower MSE than GBLUP, confirming that G × EBLUP performed better.

#### Pig and maize data

**FIGURE 3:**
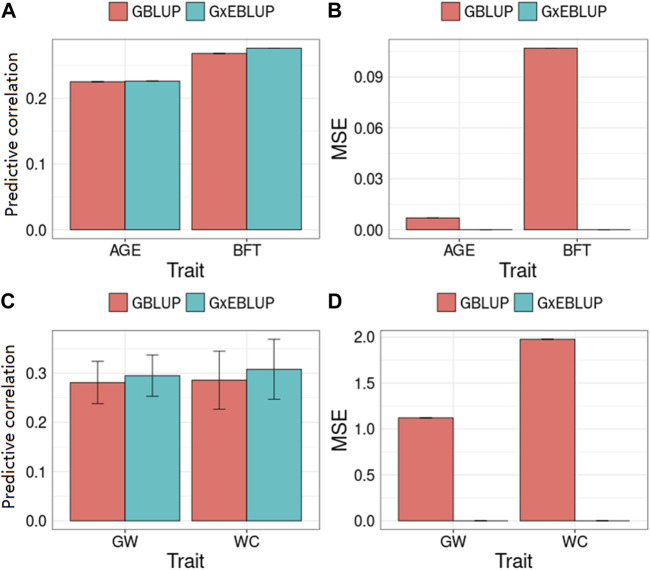
GBLUP and G × EBLUP method for the **(A,C)** accuracy and **(B,D)** mean squared error (MSE) of genomic prediction in pigs and maize. AGE: days to 100 kg; BFT: backfat thickness adjusted to 100 kg; GW: grain weight; WC: water content. MSE is a relative value by assuming that the MSE of GBLUP method is equal to 0 because of the large MSE value.

Figure 3 and Table 3 present the accuracy and MSE of genomic prediction on pig and maize data. According to the results of G × EWAS regarding AGE trait in pigs, 27,762; 14,117; 7,420; 3964 and 2,117 SNPs were selected as G × E markers in G × EBLUP, and the accuracies of G × EBLUP under different G × E markers (1-E01–1-E05) were not significantly different. The average prediction accuracy obtained using G × EBLUP was 0.225, which was same as that obtained using GBLUP. Moreover, no differences were noted in the MSE between G × EBLUP and GBLUP. These findings indicated that AGE was not affected by GEI. However, for BFT, G × EBLUP showed the best performance at the *p*-value of 1-E02, the prediction accuracy was improved by 3% compared with that of GBLUP, and the MSE was reduced by 0.107, decreasing from 2.707 to 2.600.

Compared with pig data, the genomic prediction of G × EBLUP showed considerable improvement in maize data. As shown in Table 3, for the trait GW from a randomly selected replicate, G × EBLUP showed the best performance at the *p*-value of 1-E02, the genomic prediction accuracy was improved by 6.25% compared with that of GBLUP, and MSE was reduced from 46.323 to 46.174. For the trait WC from a randomly selected replicate, the highest prediction accuracy of G × EBLUP was obtained at the *p*-value of 1-E03, the prediction accuracy was improved by 7.8% compared with that of GBLUP, and MSE was reduced from 721.588 to 720.854. The average prediction accuracy of 5 repetitions of 10-fold CV for GBLUP and G × EBLUP are shown in Figure 3, G × EBLUP showed approximately 5.0 and 7.7% improvement in prediction accuracy for GW and WC in maize population, respectively. Moreover, G × EBLUP also showed a lower prediction MSE than GBLUP, which further verified the advantages of G × EBLUP.

### Sensitive environment detection in real data

**FIGURE 4:**
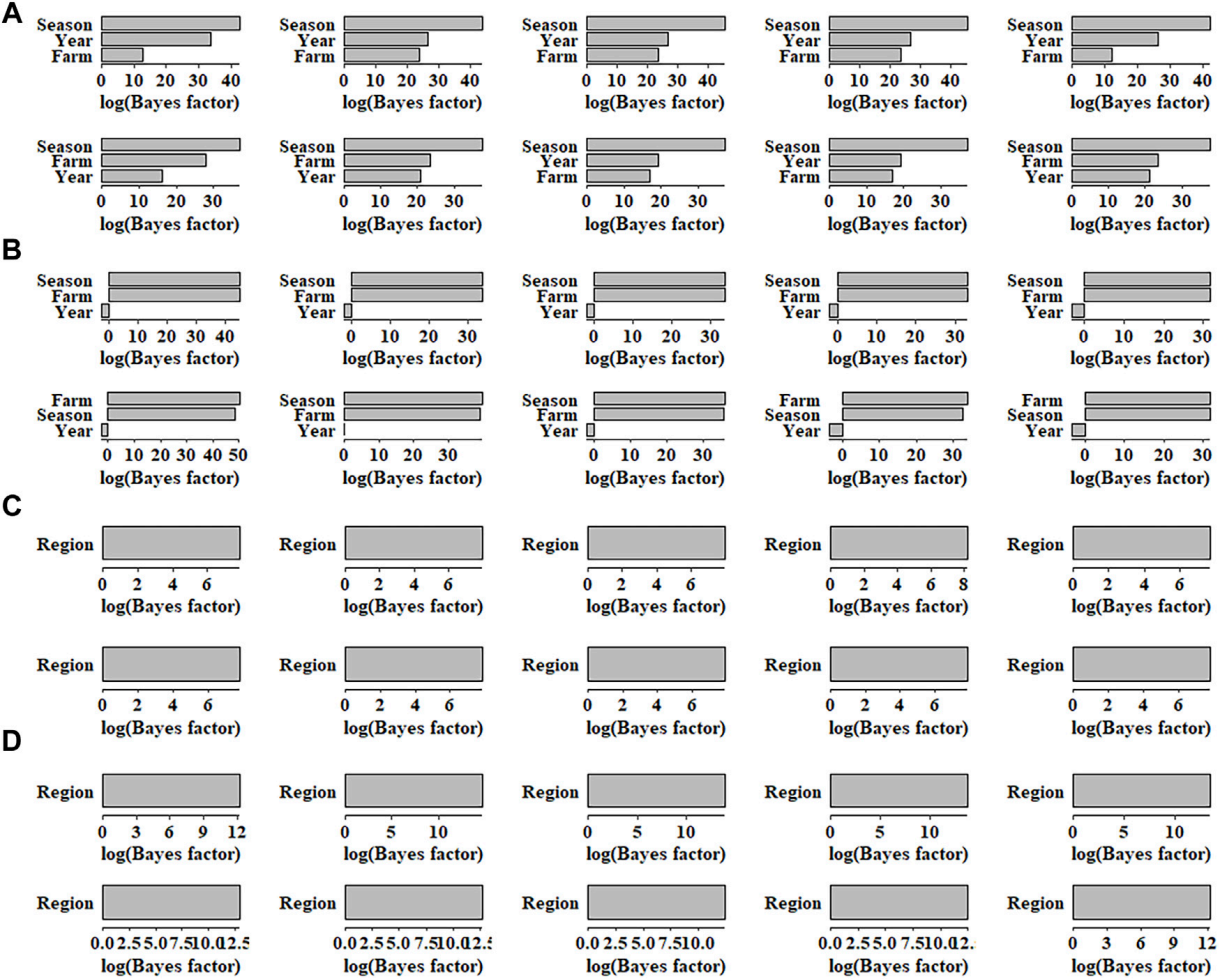
Bayes factors of 10 single nucleotide polymorphisms for **(A)** AGE, **(B)** BFT, **(C)** GW and **(D)** WC in different environmental factors. AGE: days to 100 kg; BFT: backfat thickness adjusted to 100 kg; GW: grain weight; WC: water content.

The BF for each environmental factor was obtained, and then the sensitive environments were assessed accordingly. In pig population, for AGE and BFT, 10 SNPs each with the smallest *p*-values were selected for sensitive environmental detection. As shown in Figure 4, for AGE, the top 10 SNPs regarding season showed the largest BF, indicating that the most sensitive environmental factor for the top 10 SNPs was season. Similarly, the least sensitive environmental factors for SNPs were farm and year, as the values of BF of farm and year were equally low. For BFT, the most sensitive environmental factors for all SNPs were farm and season, and their BF values were also same; year was the least sensitive environmental factor (Figure 4). In maize population, as shown in Figure 4, the averaged log BF values indicated that the region environment variable has a strong GEI (Log(BF) > 3) with WC and GW. Additionally, the BF values of WC were higher than that of GW, which was also consistent with the superiority of genomic prediction of WC (Figure 3).

### Computing time

The average computation time for G × EBLUP and GBLUP to complete each fold of CV is presented in Table 4. Running time of the methods was measured in minutes on an HP server **(**CentOS Linux 7.9.2009, 2.5 GHz Intel Xeon processor and 515G total memory). In all scenarios, G × EBLUP runs longer than GBLUP, mainly because running G × EBLUP requires concurrently running G × EWAS, which is a single marker regression model with a long running time, e.g. G × EWAS took an average of 45.8 min in each fold of CV to complete the analysis, requiring considerably less time than GBLUP (15.05 min). In addition, there is no obvious difference here between different traits in the same population due to the same size population and number of SNPs.

**TABLE 4:** Average computation time for G × EBLUP and GBLUP to complete each fold of cross-validation.

Date set	Trait^a^	G × EBLUP	GBLUP
Simulation	V0.25	45 min 48 s	15 min 3 s
	V1	46 min 27 s	15 min 12 s
	V2	46 min 13 s	15 min 9 s
Pig	AGE	30 min 9 s	2 min 14 s
	BFT	30 min 14 s	2 min 18 s
Maize	GW	26 min 13 s	1 min 7 s
	WC	26 min 8 s	1 min 10 s

a V0.25, V1 and V2: The traits with variance of G × E interactions of 0.25, 1 and 2, respectively, in simulated data.

## Discussion

G × E interactions play an important role in livestock and plants and should be considered in breeding programmes to select elite individuals in specific environments (Crossa, 2012; Heslot et al., 2014; Jarquin et al., 2017; Perez-Rodriguez et al., 2017; Tiezzi et al., 2017; Liu et al., 2019; Zhang et al., 2019; Braz et al., 2021). However, because of their complexity, G×E interactions are usually ignored in conventional breeding and the current widely used methods of estimating genomic breeding value, e.g. GBLUP (VanRaden, 2008), single-step GBLUP (Misztal et al., 2009), BayesA (Meuwissen et al., 2001), BayesCpi (Habier et al., 2011), which could lead to biases in the estimation of breeding values and selection decisions. Although the multi-trait (Richard, 1996) and reaction norm models (Rebecka et al., 2002) are two prevalent methods for handling GEI in the estimation of genomic breeding value, our previous studies have explicated the disadvantages of these two types of methods (Song et al., 2020b). Moreover, these two methods could not detect the markers associated with GEI. In this study, we proposed G × EBLUP, which is a novel method for genomic breeding value estimation that takes GEI into account. The core of G × EBLUP is the estimation of GEI using G × EWAS by including an additional per-individual effect term that accounts for GEI; it is also powerful for the identification of the SNPs that are susceptible to GEI. Comprehensive simulation studies and real data of pigs and maize have demonstrated the superiority of the proposed method.

In the simulated data, our results indicated the superiority of the G × EBLUP method for genomic prediction in all scenarios, which was more remarkable when the variance of GEI was large (Figures 2A,B), showing that the new method can appropriately handle GEI. Our results also showed that there was no significant difference in the prediction accuracy of G × EBLUP under different numbers of environment variables (Figures 2C,D), implying that the number of environment levels have no effect on our new method. This is a major highlight of the G × EBLUP method compared with the methods based on the multi-trait (Richard, 1996) and reaction norm models (Rebecka et al., 2002), in which the number of environment levels was the main limitation (Song et al., 2020a; Song et al., 2020b). The advantage of G × EBLUP for joint G × E analysis of multiple environment variables could be that multiple environments can interact with a single genetic locus to influence the phenotypes (Moore et al., 2019). In our new method, the interactions of genotype with different environments could be represented by one or more markers, as explicated by G × EWAS. Therefore, it was not sensitive to the number of environment levels.

In this study, we proposed G × EWAS and a score-test statistic to identify the significance of SNP affected by GEI; the details of G × EWAS and its computational complexity can be found in Additional File 1 Supplementary Material. In G × EWAS, E is the N×L matrix of L observed environments, and ${EE}^{\prime }$ is used as a variance–covariance structure for G × E effects; thus, G × EWAS is a single-SNP regression model, which is different from the multi-trait (Richard, 1996) and reaction norm models (Rebecka et al., 2002). The linear covariance function (**EE’**) was primarily used because of two appealing properties. First, as the number of samples typically exceeds the number of environments in larger populations (L << N), a low-rank linear covariance is noted, which enables parameter inference with a computational complexity that scales linearly with the increasing population size. Second, a linear covariance is directly interpretable, as there is one-to-one correspondence between G × EWAS and linear regression using L covariates to account for GEI. Compared with the four methods proposed by Wang et al. (2019), our results showed the obvious advantages of G × EWAS in GEI detection (Additional File 3: Supplementary Figures S8–S10 and Table 2). The low efficiency of the other methods could be because the selection, epistasis and phantom vQTL can also cause vQTL instead of just GEI, which may lead to biases in the detection of G × E markers, e.g. the overlapped simulated QTLs were decreased for L-mean and L-median when the variance of G × E increased. Although the efficiency of G × EWAS was improved with increase in the variance of GEI, it yielded higher number of overlap QTLs with GEI. As a combination of the standard linear mixed model for genome-wide association study and StructLMM, our proposed G × EWAS performed better than StructLMM (Additional File 3: Supplementary Figures S8–S10 and Table 2). The superiority of G × EWAS was mainly because it built a genomic relationship matrix to capture the realised relationships among individuals; moreover, it can accurately capture the effect of each environment on markers by adding the GEI vector in the model, which follows a multivariate normal distribution. In the scenario of larger variance of GEI, more QTLs would contribute to the GEI, increasing the weight of per-individual effect size as described in Eq. 1; thus, it could be easily detected using G × EWAS.

Although G × EWAS could detect more significant SNPs associated with GEI using Bonferroni correction, only a small amount of the whole markers were detected. The best performance of G × EBLUP was obtained at the marker selection criterion of *p*-value of E02 (BFT in pig and WC in maize) or E03 (simulated data and WC in maize) (*p*-values < 10^–2^ or 10^–3^) in all scenarios (Table 3). In fact, the performance of G × EBLUP at E02 and E03 was similar. Accordingly, the selected SNPs with GEI for simulated data, AGE and BFT in pig and WC and GW in maize were enough to build a genomic relationship matrix to elucidate the contribution of GEI. The number of selected SNPs with GEI in G × EBLUP was lower than that of the significant SNPs at false discovery rate of 0.05 (Additional File 2: Supplementary Table S2). Therefore, it is reasonable to use *p*-values of <10^–3^ as threshold for determination of SNPs with GEI in G × EBLUP. The advantage of G × EBLUP over GBLUP is mainly because G × EBLUP allows the assignment of different weights to the genomic variants in the different genomic relationship based on their estimated genomic parameters, which can better fit the genetic architecture of the trait, while randomly selected a subset of SNPs that are not all associated with the trait, giving more weight to these SNPs in G × EBLUP does not improve the accuracy of genomic prediction (results not shown).

Along with the mapping of G × E markers, G × EWAS could determine favourable environment using BF of SNPs on each environment. This is extremely helpful for market-specific breeding in animals and plants as it may provide further explanation to those individuals who have a higher risk of being affected by GEI in a certain environment variable (sensitive environment). In this study, farm was identified as a sensitive environment for BFT in pigs, which allows the selection of elite individuals with good performance in specified farms. Further, our results showed that GEI was different for distinct traits, e.g. similar genomic prediction accuracies were obtained for G × EBLUP and GBLUP for AGE, whereas G × EBLUP showed improvement by approximately 3% in the prediction accuracy for BFT in pigs (Figure 3). This observation is consistent with that of our previous report that showed GEI for BFT but not for AGE (Song et al., 2020b). This could be explained by values of the variance of the slope ( $\sigma_{a_{1}}^{2}$ ) compared with those of the intercept ( $\sigma_{a_{0}}^{2}$ ) in the reaction norm model; $\sigma_{a_{1}}^{2}$ / $\sigma_{a_{0}}^{2}$ were 0.002 and 0.348 for AGE and BFT, respectively. Thus, traits with small variance of GEI cannot improve the accuracy of genomic prediction using G × EBLUP even after the identification of more significant SNPs on AGE. Similarly, the less significant SNPs in the maize data showed larger variance and greater improvement in the genomic prediction accuracy than those in the pig data (Figure 3). Further, this might be due to the trait characteristics of plants, which are more vulnerable to GEI than livestock, the effect of environment in animal become ignorable as the industrial management. Conversely, the small sample size of the maize may have reduced the power of G × EWAS, leading to the identification of a small number of significant GEI markers, which may explain why the gain of G × EBLUP was lower than expected. In addition, using sensitive tests to detect sensitive environments is an alternative, and then fitting the overlap environment of SNPs in the G × EBLUP model based on the pre-defined environment. However, the effect of this method and how to fit it in G × EBLUP model need to be investigated in the future.

Our results showed G × EBLUP is a powerful alternative to the conventional method for the estimation of genomic breeding value. However, there are several limitations in this approach. First, G × EWAS is not a whole-genome regression model and does not account for the genome-wide contribution of all other variants, thus G × EWAS is still a single marker regression model with a long calculation time (Table 4). G × EWAS assumes all SNPs affected by GEI in the current model, and it could not differentiate between the significant SNPs with or without GEI. It might be the reason why a large number of significant SNPs were detected in the simulated data, although only few overlapped with simulated QTLs. The same phenomenon was also found in other methods, such as Bartlett, F-Killeen, L-mean, L-median and StructLMM, implying that the detection of GEI is more difficult due to the flexibility of the environment. Second, although sensitive environment can be obtained by calculating the BF value for each environment variable, the BF value of each level of an environment variable cannot be obtained (e.g., BF for each farm in pig data), which might be important for directional breeding. Third, G × EBLUP has an advantage only when the variance of the G × E interaction is relatively large, e.g., similar genomic prediction accuracies were obtained for G × EBLUP and GBLUP for AGE, as was not affected by GEI. Finally, G × EWAS cannot handle binary traits at present, as G×E tests need the estimation of nuisance parameters to capture the main effects of binary traits, and estimating these parameters requires high-dimensional integration and the inversion of a high-dimensional similarity matrix. Nevertheless, it is worth being investigated in the future.

Moreover, G × EBLUP can be extended to single-step method (ssG × EBLUP), which could improve the genomic prediction accuracy using pedigree and genomic information (Misztal et al., 2009; Aguilar et al., 2010; Christensen and Lund., 2010).

## Conclusion

The G × EBLUP method proposed in this study showed the following four features: 1) genomic prediction was performed using the G × EBLUP method by considering GEI and yielded higher accuracies and lower MSE in both simulated and real pig and maize data when the variance of G × E interaction is large; 2) it could powerfully detect the genome-wide SNPs subject to GEI; 3) the number of environment levels did not influence the genomic prediction accuracy of the proposed G × EBLUP, circumventing the limitation of current methods; 4) it could determine favourable environment using SNP BF for each environment, thus being useful for market-specific animal and plant breeding.

## Data Availability

The simulated data, pig and maize data supporting the conclusions of this article are available from Figshare: https://figshare.com/articles/dataset/GXEBLUP/20347368.
