# Supplementary material for: G × EBLUP: A novel method for exploring genotype by environment interactions and genomic prediction
Source: Front Genet. 2022 Sep 12;13:972557. doi: 10.3389/fgene.2022.972557 (PMC9510768; doi:10.3389/fgene.2022.972557)
Supplement: Supplementary file 1 [file DataSheet3.docx]

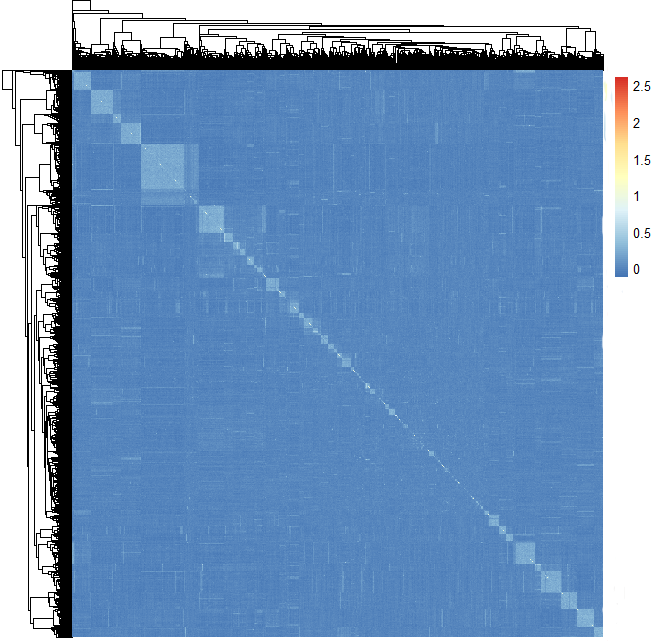


Figure S1. Heat map of genomic relationship matrix of 7334 Chinese Holstein dairy cattle


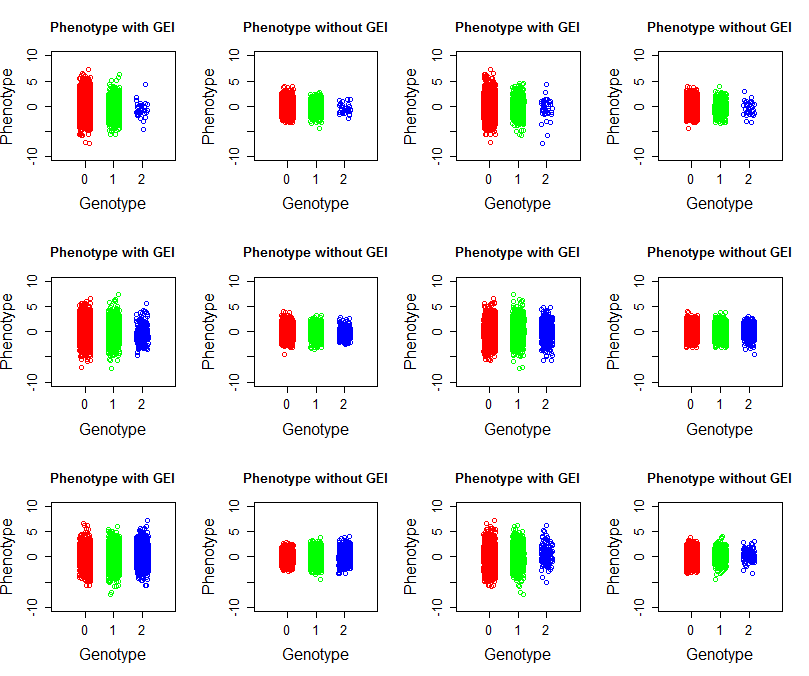


Figure S2. Phenotypic variation of each genotype of 6 SNPs with or without G × E interaction at G × E interaction variances with 0.25.


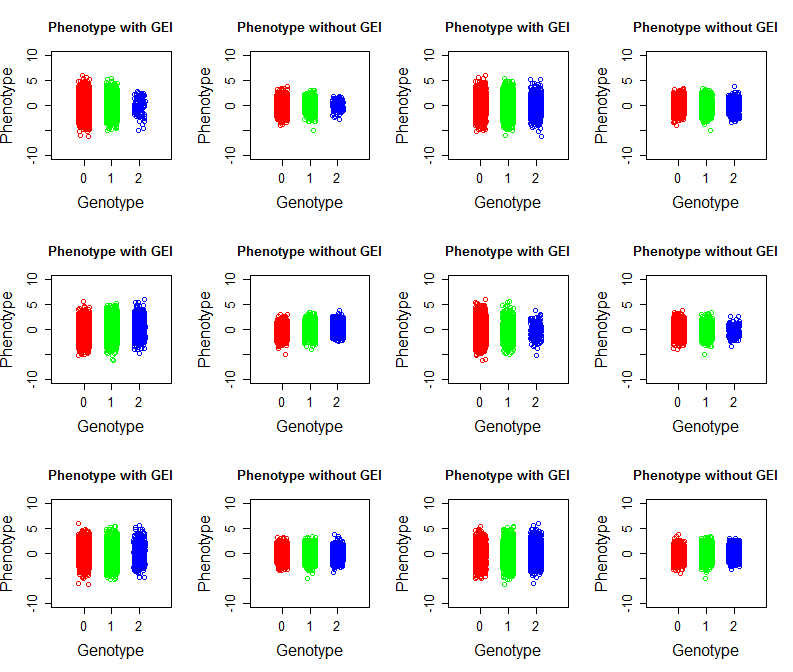


Figure S3. Phenotypic variation of each genotype of the 6 SNPs with or without G × E interaction at G × E interaction variances with 0.25 and 2 covariates in simulated data.


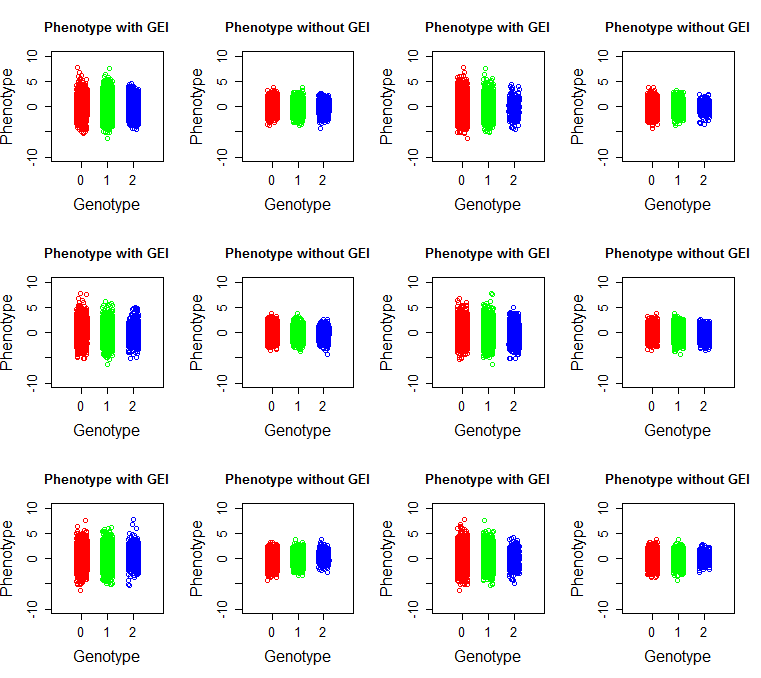


Figure S4. Phenotypic variation of each genotype of the 6 SNPs with or without G × E interaction at G × E interaction variances with 0.25 and 3 covariates in simulated data.


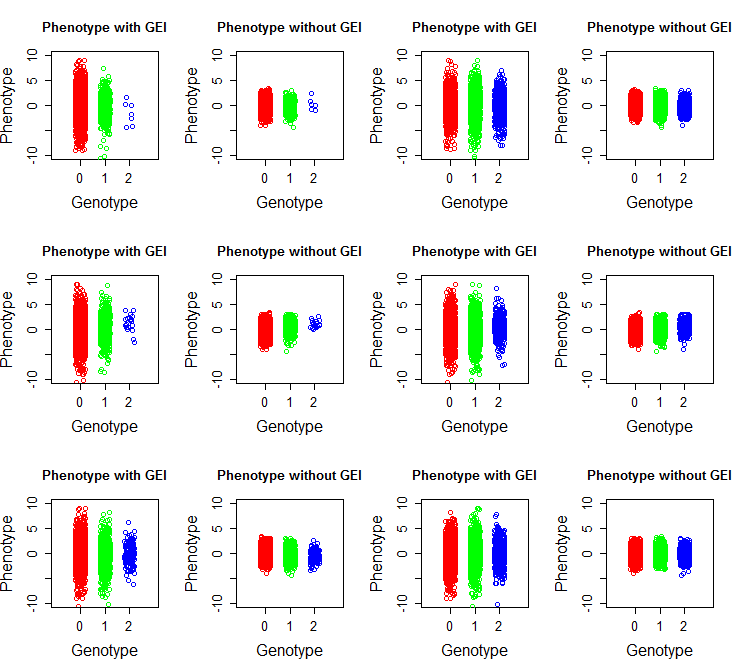


Figure S5. Phenotypic variation of each genotype of 6 SNPs with or without G × E interaction at G × E interaction variances with 1.


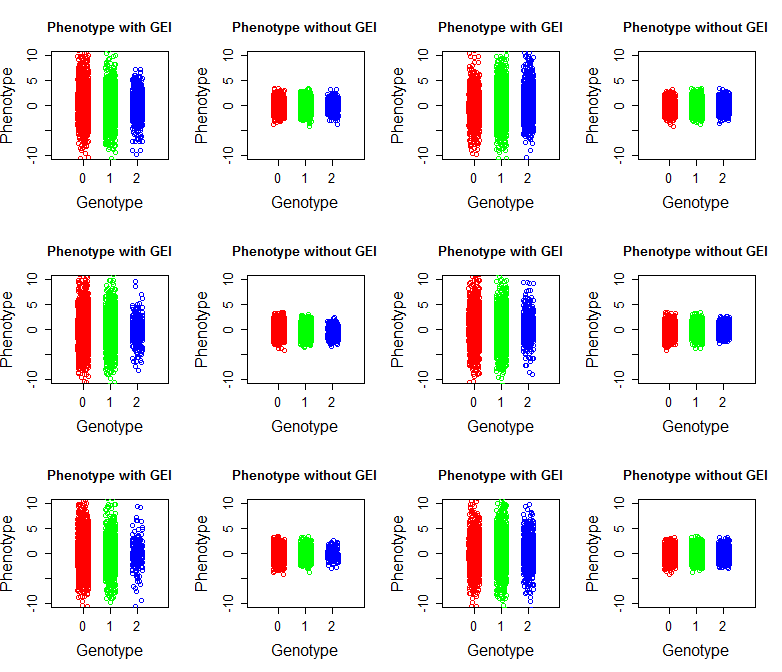


Figure S6. Phenotypic variation of each genotype of 6 SNPs with or without G × E interaction at G × E interaction variances with 2.

.


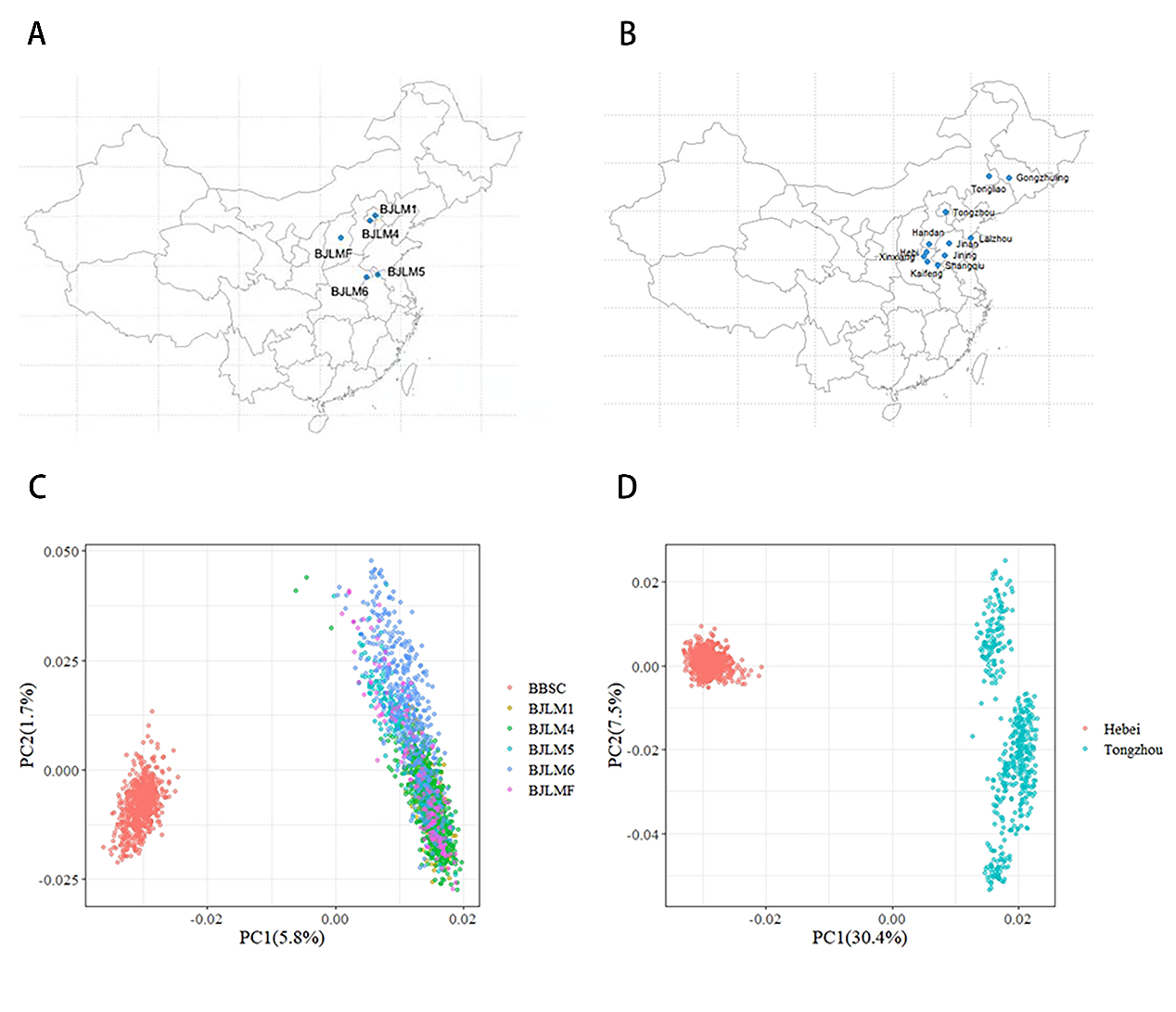


Figure S7. The location of five pig breeding farms (A) and eleven planting regions of maize (B), and principal component analysis (PCA) for (C) pig and (D) maize. BBSC represents another Yorkshire population; Tongzhou represents population from 11 maize planting regions in (B), and Hebei represents another maize population from Hebei.


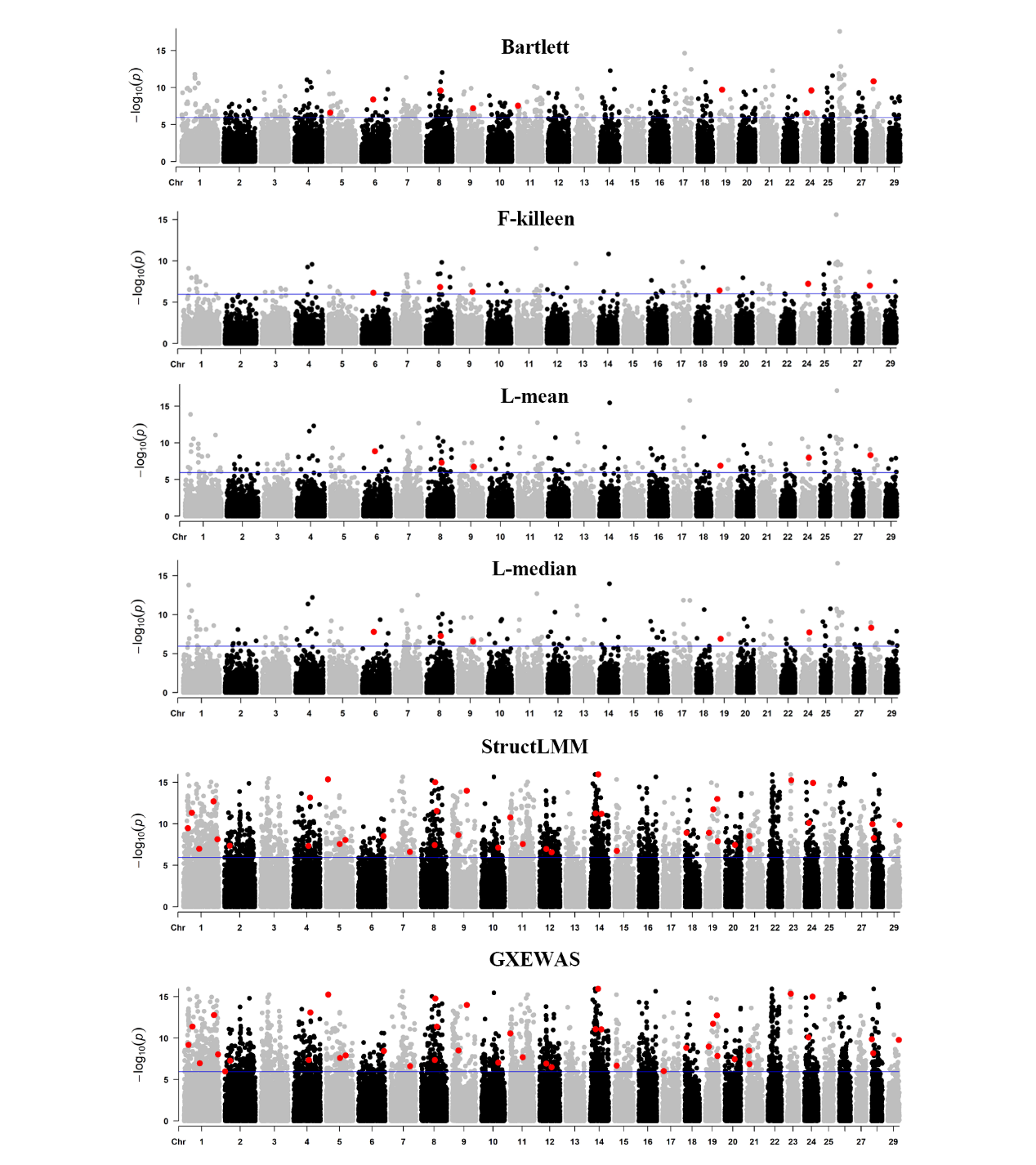


Figure S8. G × E marker genome-wide association analysis by G × EWAS and other five approaches at G × E interaction variances with 0.25 in simulated data. Red dots represent the G × E interaction QTL. The blue line indicates the Bonferroni correction as the threshold.


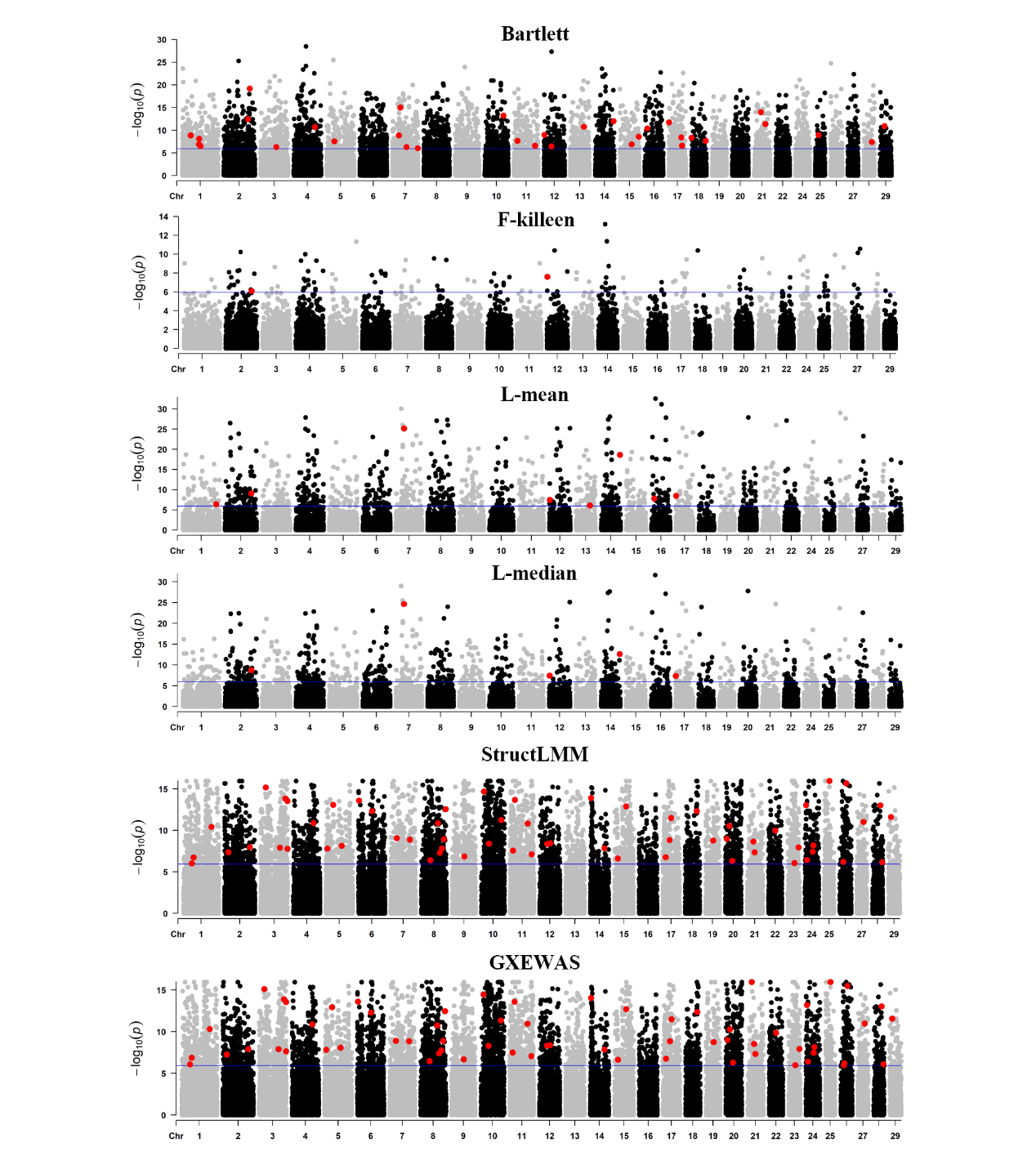


Figure S9. G × E marker genome-wide association analysis by G × EWAS and other five approaches at G × E interaction variances with 1 in simulated data. Red dots represent the G × E interaction QTL. The blue line indicates the Bonferroni correction as the threshold.


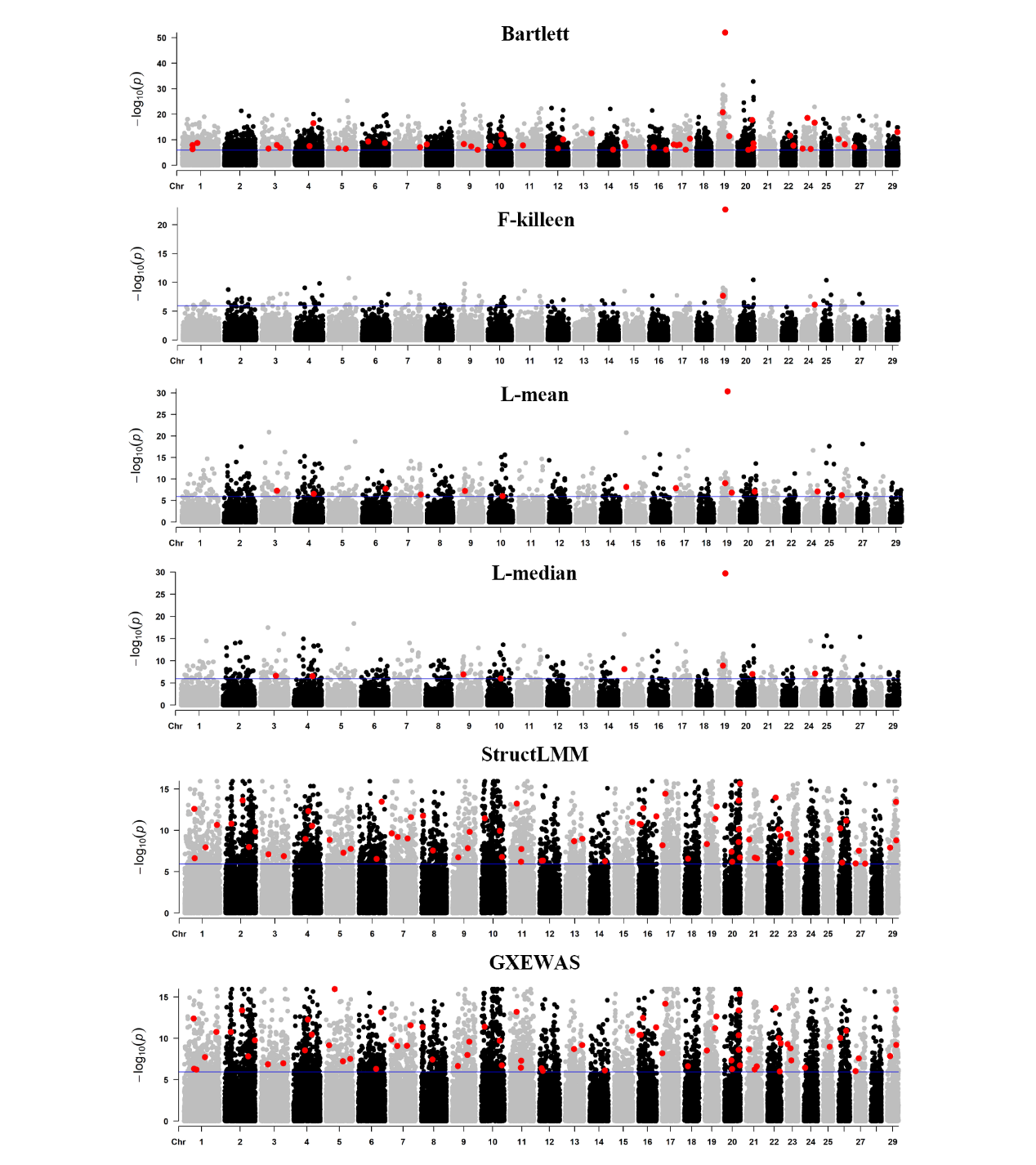


Figure S10. G × E marker genome-wide association analysis by G × EWAS and other five approaches at G × E interaction variances with 2 in simulated data. Red dots represent the G × E interaction QTL. The blue line indicates the Bonferroni correction as the threshold.


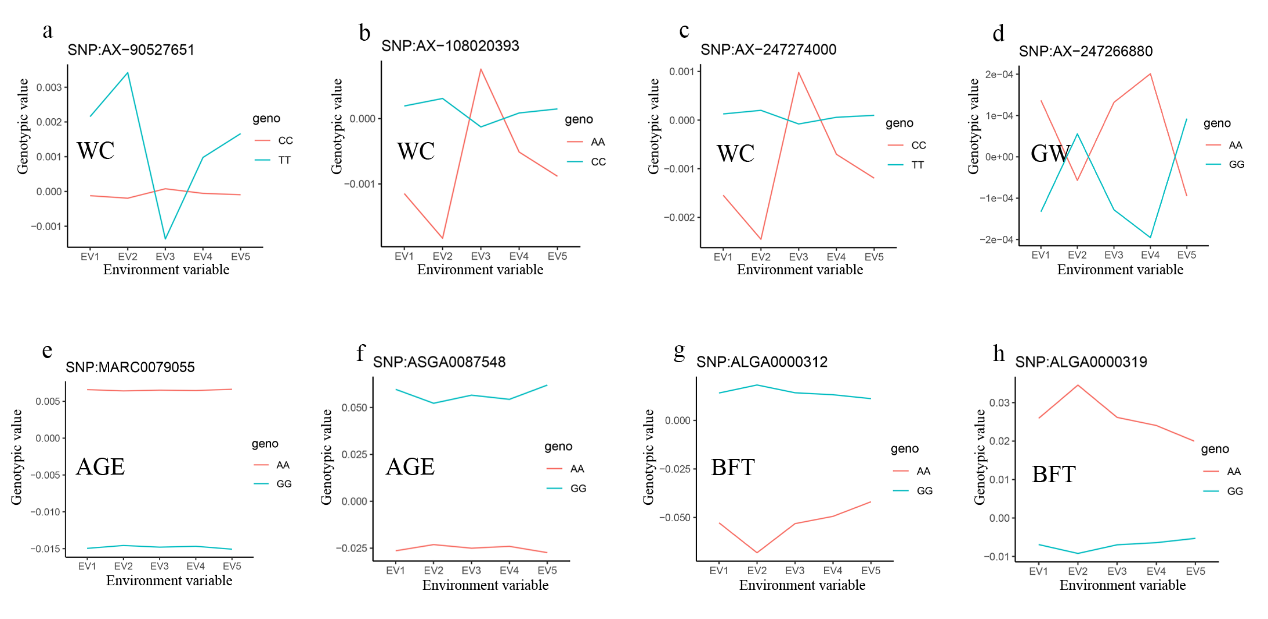


Figure S11 Genotypic values of significant single nucleotide polymorphisms with G × E interactions associated with (a–c) WC, (d) GW, (e and f) AGE and (g and h) BFT at different environment variables in pigs and maize. WC, water content; GW, grain weight; AGE, days to 100 kg; BFT, backfat thickness adjusted to 100 kg


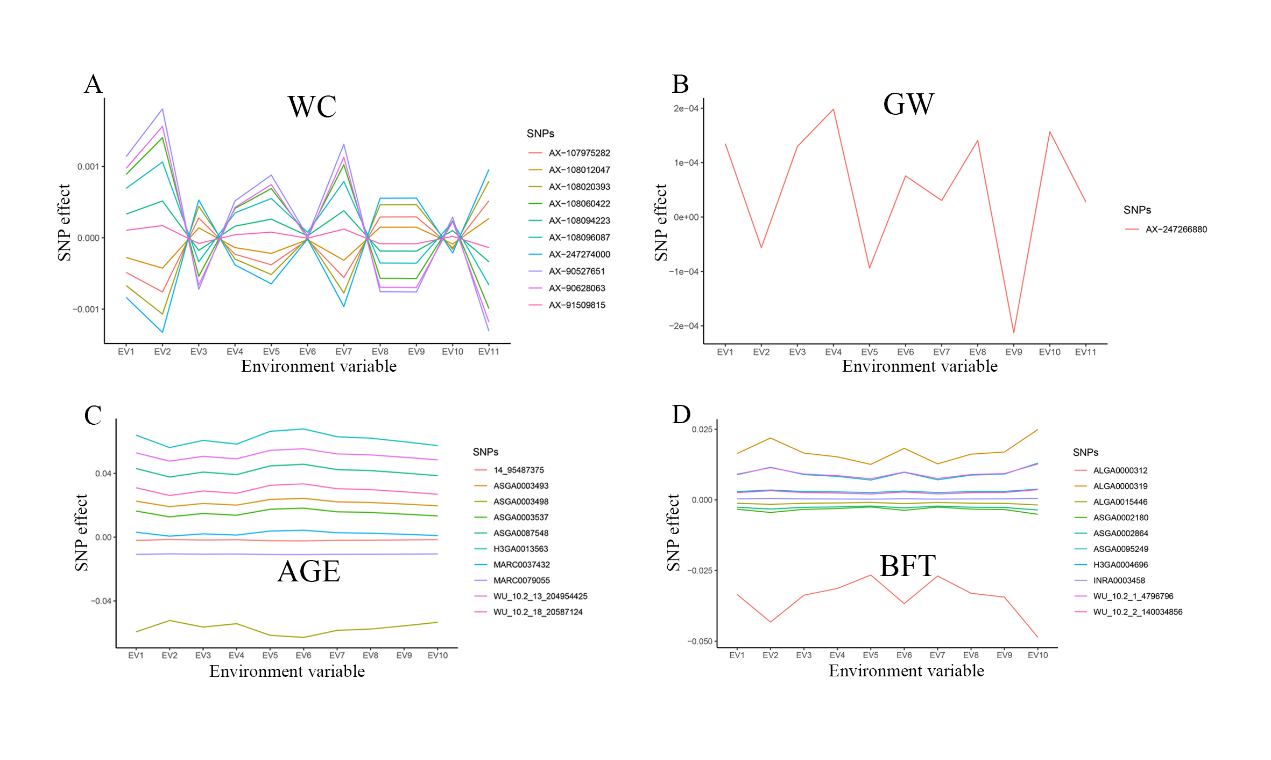


Figure S12. Estimated effects of significant SNPs with G × E interactions associated with (A) WC, (B) GW, (C) AGE and (D) BFT at different environmental variables in maize and pig. WC, water content; GW, grain weight; AGE, days to 100 kg; BFT, backfat thickness adjusted to 100 kg.
