# Supplementary material for: G × EBLUP: A novel method for exploring genotype by environment interactions and genomic prediction
Source: Front Genet. 2022 Sep 12;13:972557. doi: 10.3389/fgene.2022.972557 (PMC9510768; doi:10.3389/fgene.2022.972557)
Supplement: Supplementary file 2 [file DataSheet2.docx]

Table S1. The assigned and estimated variance of additive effect, G × E interaction effect and residual in 20 replicates of simulated data.

| Simulated scenarios | Parameter | Assigned | Estimates |
| --- | --- | --- | --- |
| Var($\alpha_{1}$)=0.25 and N-Env=1 | Var($\alpha_{0}$) | 1 | 0.976±0.061 |
|  | Var($\alpha_{1}$) | 0.25 | 0.258±0.024 |
|  | Var($e_{0}$) | 1 | 1.019±0.030 |
| Var($\alpha_{1}$)=0.25 and N-Env=2 | Var($\alpha_{0}$) | 1 | 0.965±0.056 |
|  | Var($\alpha_{1}$) | 0.25 | 0.256±0.018 |
|  | Var($e_{0}$) | 1 | 1.008±0.032 |
| Var($\alpha_{1}$)=0.25 and N-Env=3 | Var($\alpha_{0}$) | 1 | 0.978±0.063 |
|  | Var($\alpha_{1}$) | 0.25 | 0.254±0.022 |
|  | Var($e_{0}$) | 1 | 1.009±0.032 |
| Var($\alpha_{1}$)=1 and N-Env=1 | Var($\alpha_{0}$) | 1 | 0.958±0.059 |
|  | Var($\alpha_{1}$) | 1 | 1.084±0.061 |
|  | Var($e_{0}$) | 1 | 1.000±0.029 |
| Var($\alpha_{1}$)=2 and N-Env=1 | Var($\alpha_{0}$) | 1 | 0.946±0.052 |
|  | Var($\alpha_{1}$) | 2 | 2.093±0.102 |
|  | Var($e_{0}$) | 1 | 1.015±0.042 |

Assigned: parameters set in the simulated program; Estimates: estimated by using a reaction norm model with genomic information; N-Env: Number of covariate of environment; Var($\alpha_{0}$), Var($\alpha_{1}$) and Var($e_{0}$): Variances of additive effect, G × E interaction effect and residual.

Table S2. Significant SNPs of genotype by enviroment interactions detected in simulated data and real data of pig and maize at false discovery rate of 0.05 and 0.01

| Data | Trait^a^ | 0.05 | 0.01 |
| --- | --- | --- | --- |
| Simulated data |  | 17,500 | 11,808 |
| Pig | AGE | 17,602 | 9866 |
|  | BFT | 30,496 | 22,118 |
| Maize | GW | 2958 | 425 |
|  | WC | 18,688 | 8663 |

^a^AGE: days to 100 kg; BFT: backfat thickness adjusted to 100 kg; GW: grain weight; WC: water content.
