# Supplementary material for: G × EBLUP: A novel method for exploring genotype by environment interactions and genomic prediction
Source: Front Genet. 2022 Sep 12;13:972557. doi: 10.3389/fgene.2022.972557 (PMC9510768; doi:10.3389/fgene.2022.972557)
Supplement: Supplementary file 3 [file DataSheet1.docx]

**Supplementary Methods**

*Computational complexity*

To evaluate the interaction test of G × EWAS, we need to first fit the null model and then compute the corresponding score test statistics. A naive implementation of both of these operations would result in computations that scale cubically with the number of individuals, i.e.$O(N^{3})$, $N$ is the number of individuals. When considering a linear covariance of genomic relationship matrix, the total covariance matrix is $\sigma_{g}^{2}\mathbf{G}$**+**$\sigma_{e}^{2}\mathbf{I}$. In this setting, the null model can be fitted with computational complexity $O({NR}^{2}+R^{3})$, where $R$ is the rank of the first covariance term (in this case, *R=N*). We refer to Lippert et al. (2014) and Casale et al. (2015) for further details.

Concerning the computation of the score test statistics for interaction test, Q can be calculated with computational complexity$O({NL}^{2}+{{NK}^{2}+NLK+L^{2}K+K}^{3}+L^{3})$, where $K$ corresponds to the number of covariates, while the coefficient $\mathbf{a}$ of $\chi^{2}$ mixture (eigenvalues of $\mathbf{P}^{\frac{\mathbf{T}}{\mathbf{2}}}\mathbf{K}_{\mathbf{1}}\mathbf{P}^{\frac{\mathbf{1}}{\mathbf{2}}}$), can be computed in $O({NL}^{2}+{{NK}^{2}+NLK+L^{2}K+K}^{3}+L^{3})$. The computation of P values either using the Davies (1980) or Liu (2008) method does not depend on the number of individuals.

Finally, the one-dimensional numerical integration step (using Davies or Liu method) has a computational complexity that is independent of the number of individuals.

**References**

Casale, F. P., Rakitsch, B., Lippert, C., Stegle, O. (2015). Efficient set tests for the genetic analysis of correlated traits. [Journal Article; Research Support, N.I.H., Extramural]. *Nat. Methods*. 12(8), 755-758. doi: 10.1038/nmeth.3439

Davies, R. (1980). Algorithm as 155: The Distribution of a Linear Combination of χ2 Random Variables. *Journal of the Royal Statistical Society. Series C (Applied Statistics)*. 29(3), 323-333

Huan, L., Yongqiang, T., Hao, H. Z. (2008). A new chi-square approximation to the distribution of non-negative definite quadratic forms in non-central normal variables. *Computational Statistics and Data Analysis*. 53(4)

Lippert, C., Xiang, J., Horta, D., Widmer, C., Kadie, C., and Heckerman, D., et al. (2014). Greater power and computational efficiency for kernel-based association testing of sets of genetic variants. [Journal Article; Research Support, N.I.H., Extramural; Research Support, Non-U.S. Gov't]. *Bioinformatics*. 30(22), 3206-3214. doi: 10.1093/bioinformatics/btu504
